# Behavioral interventions for coronary heart disease patients

**DOI:** 10.1186/1751-0759-6-5

**Published:** 2012-02-02

**Authors:** Kristina Orth-Gomér

**Affiliations:** 1 Dept of Clinical Neuroscience, Karolinska Institutet, Stockholm Sweden

## Abstract

**Introduction:**

There is a strong clinical need to provide effective stress reduction programs for patients with an acute coronary syndrome. Such programs for men have been implemented and their cardiovascular health benefit documented. For women such programs are scarce.

In this report, The feasibility of a cognitive method that was recently demonstrated to prolong lives of women is tested. A setting with gender segregated groups was applied.

**Method:**

The principles of a behavioural health educational program originally designed to attenuate the stress of patients with coronary prone behaviours were used as a basis for the intervention method. For the groups of female patients this method was tailored according to female stressors and for the groups of men according to male stressors. The same core stress reduction program was used for women and men, but the contents of discussions and responses to the pre planned program varied. These were continuously monitored throughout the fifteen sessions. Implementation group: Thirty consecutive patients, eleven women and nineteen men, hospitalized for an acute coronary syndrome were included in this intervention. All expressed their need to learn how to cope with stress in daily life and were highly motivated. Five groups, three groups of men and two groups of women were formed. Psychological assessments were made immediately before and after completion of the program.

**Results:**

No gender differences in the pre planned programs were found, but discussion styles varied between the women and men, Women were more open and more personal. Family issues were more frequent than job issues, although all women were employed outside their homes. Men talked about concrete and practical things, mostly about their jobs, and not directly about their feelings. Daily stresses of life decreased significantly for both men and women, but more so for women. Depressive thoughts were low at baseline, and there was no change over time. In contrast, anxiety scores were high at baseline and decreased significantly, but more so for women than for men.

**Conclusion:**

Women are likely to benefit from women's groups. Men may prefer to have one or two women in the group, but women fare better in gender segregated groups.

## Introduction

Cardiovascular disease (CVD) is our most important cause of death, with about four million deaths in Europe and about 50 000 deaths every year in Sweden. It is also a frequent cause of disability and of repeated hospitalizations. The burden on society is important, as is the burden on the patient. Although female patients die as frequently as males from CVD, the scientific emphasis has been on male patients, whereas research in women has been neglected.

Rehabilitation of patients with CVD has traditionally focused upon physical capacity and physical exercise, and has been mostly conducted in men. We have recently shown that a stress reduction program, specifically tailored to women's needs and stress profiles, may be beneficial and even reduce mortality in women with CHD. To improve physical capacity is as important for men as for women, but many other difficulties also face the CVD patient. The strains and stresses of daily life are complex and demanding for both men and women, and extend beyond the need to be physically fit. Anxiety and depressive thoughts are common in patients with CVD, and behavioral interventions addressing these issues have been developed to help patients meet the challenges of every day life. The importance of these issues, along with scientific evidence and methodological considerations were addressed in detail in the clinical guidelines for CVD prevention of the European Society of Cardiology [1].

Among current behavioral intervention programs [2-4], few have been systematically tested concerning their effectiveness for both men and women.

In most studies, health benefits have been found among men but rarely among women with CHD. The Recurrent Coronary Prevention Project (RCPP) [5] used a program that was originally designed to attenuate the coronary prone type A behavior of men and a modified version was implemented for use with female Swedish patients [6]. This program has been applied to women living in rural [6] and urban settings[7,8]. Health benefits were found for short term subjective stress [9] as well as for long term survival [8].

A modified version of this program has now been streamlined for both men and women. In this feasibility report we describe the modified version of the program, examine its adequacy for use with men and women, and evaluate the similarity of its impact on health parameters of men and women. We have included both male and female patients, but maintained gender specific groups in order to investigate the applicability of the program for both sexes.

## Material and methods

Patients were obtained from the "heart school", from the outpatient cardiology consultation clinic, and from doctors and other care givers who identified patients who were in need of stress reduction.

The need to be present at all sessions and the evaluation with self administered questionnaires were explained. The relevant questionnaires were distributed, filled out and collected prior to the first session. At the last meeting all questionnaires were distributed again together with an evaluation form, in which the participants could express in their own words how they had experienced the sessions.

All patients were also invited to a personal cardiovascular health check/consultation at two occasions; at the midpoint and at the end of the course.

### Recruitment of patients

The intervention groups focusing on cardiac patients were formed in a Stockholm community Hospital. Two criteria for being selected to join a group had to be met:

1) Post AMI, PTCI or CABG, and

2) Self experienced stress problems.

We recruited 21 male and 13 female patients during one year; all aged 70 years or less (mean 55.5 years). All subjects had suffered an acute myocardial infarction (AMI), or undergone CABG or PTCA and also reported high psychosocial stress.

Patients were divided into groups with 5-7 subjects in each group. Four groups consisted of only men and two groups of only women.

### Intervention program

The program schedule was planned for 10 original sessions, which were followed by up to ten booster sessions held to enhance practice and skills.

At the first meeting rules were set for the group sessions.

Everything that was said within the group should be considered as strictly confidential and must not be communicated to anyone outside the group. The importance of being a participant and show each other respect, was emphasized.

Each group session lasted for two hours. One of the groups chose to have a short break at the midpoint of the sessions; the others did not. Each session started with coffee and fruit.

The group leader planned and conducted the meetings and divided the time between the group members so that every participant would get an opportunity to speak at each session.

Relaxation exercises to music ended the meetings. The participants were taught techniques for relaxation; most commonly progressive muscle relaxation techniques were used.

Each session focused on a special subject (see below) and was accompanied by relevant home work tasks. At the beginning of the following session the homework from the previous session was reviewed. One such homework task could require that the participant was to observe and record his/her stress behavior-pattern during a whole week.

In one of the exercises the patient was asked to describe a situation which had provoked anger and describe it to the group. The patient's stressful and hostile responses were identified and discussed. Cognitive behavioral strategies were used in an attempt to alter the participant's stressful and angry responses. One strategy, "the Hook", had been specially designed to attenuate irritation and anger. Anger could be triggered by trivial events, for which the exercise was perceived as particularly helpful and repeatedly used. "The Hook" emphasized the opportunity of each individual to make a choice about how to react in a certain situation. The patient was asked to imagine being a fish and to experience the stressor as a "hook". He/she was then given the choice either to "bite", in the sense of getting irritated or angry, or to refrain from biting. Participants were asked to go home and exercise how to avoid "biting the hooks". At the next session they were to report about their experiences.

In another exercise, behavioral strategies for problem-solving were used in situations, which were perceived and described as threatening. The actual problem was observed, discussed and re-formulated by the group and the patient concerned; the other participants were active in giving suggestions to solutions. These were evaluated and judged by the patient who had presented the problem. Typically, the various members of the group offered different approaches to solve the problem. This approach frequently had a positive effect on the well-being and mood of the troubled patient. He/she had several approaches to choose from and could discuss these with the other group members.

It was important, however, to maintain a certain order and discipline during the sessions. Each patient was asked to speak at the order of priority - and the others were expected to listen and be supportive.

### Topics discussed at the group sessions

1. Introduction and presentation of group members and their heart condition.

2. Atherosclerosis, coronary risk factors, and psychological consequences in relation to the cardiac event

3. Psychosocial stress and physiological stress reactions

4. Individual assessment of standard coronary risk-factors - and identification of the problem areas of the group

5. Anger and hostility in response to daily stress exposure, problem-solving and cognitive strategies, e.g. the "hook", as described above.

6. Coronary prone stress behavior. Reviewing and testing patients individual stress behavior. A video is used to identify this behavior.

7. Worry, depression, anxieties, low-spiritedness, type D-behavior (depression and social inhibition). Communication training - improving communication skills by passive and active approach.

8. Every day conflict situations. The patients are introduced to examples of conflict situations and asked to deal with them. True examples from the patients' daily lives are used along with fiction.

9. Positive and negative emotions. An exercise-book with daily, concrete cognitive exercises is started and maintained throughout the course.

10. Daily practice of relaxed behavior. Walking slowly, choosing the longest queue in the grocery store, driving in the right lane without unnecessary passing in the left lane, keep smiling at other people. The patients are expected to report back about the results to the group.

11. Patterns/roles of life, roles of social relations, strong and weak "legs" to stand on - "legs" symbolized for example "mother", "daughter", "professional pride" etc. Patients were asked to focus on their strong legs.

12. Stress and personality. Describing one's strengths in the working situation, and how this strength becomes visible within professional life. Exercises to increase one's understanding how other people function and think to recognize different strengths. To some details are more important; to others the whole context is of greater significance. This can cause conflicts both at work but privately. If a person ends up in the "wrong" professional place, e.g. is forced to deal with detailed questions, although his/her talent is more appropriate for global tasks, stress and "burn-out" may be the result.

13. Defining one's own life-situation; "How is my situation now?", "How would I like it to be?", "How do I divide my time between work, leisure, friends and family?", "How would I like this distribution of time to be?", "How much time do I get for myself?", and "What is a good balance between these different life domains?"

14 - 20. Maintenance of knowledge and skills by repetition and practice.

### Psychosocial evaluation

#### Health related Quality of life

To measure the health related global life quality, the "ladder of life" was used. This is a ladder with ten steps, the lowest one illustrating the worst state of life and the highest illustrating the best. The subjects were first asked to rate their present health related quality of life, then to estimate what it was like a year ago and last, what it would be like a year from now. This measure was based on our experience with patients and developed in the early phases of the Stockholm Female Coronary Risk Study [7].

#### Sense of Coherence

The Sense of Coherence (SOC) scale was used to estimate the ability to master and cope with stressors. It describes the extent to which a person feels in tune with society around him/her and feels incorporated in a benevolent setting and system [10]. Examples of questions are "Do you have a feeling that you have been treated wrongly?" "Has it happened that people you trust have disappointed you?". Response alternatives go from 1 to 7 with a maximum score of 91 points and a minimum of 13 points. A higher SOC score corresponds to better coping capacity of the patient.

#### Anxiety and Depression

To assess subjects anxiety and depression symptoms, the Hospital Anxiety and Depression scale (HADS) [11] was used. The scale is divided into two subscales, anxiety and depression, and both consist of seven questions. All questions were scored 0,1,2,3, resulting in a maximum of 23 points and minimum of 0 on each of the scales.

#### Daily life stresses

A patient with coronary prone behavior is competitive, impatient, aggressive and in a hurry. Such a person also has a desire to achieve recognition, another highly estimated quality in modern society. To measure these stresses the "every day stress scale" was used [12]. This scale is closely related to the early Type A assessments. The Stress questionnaire consists of 20 statements, such as "I get irritated by other drivers" and "I compete with myself and others". A four point Likert scale is used, with high scores corresponding to high stress. The scale has a maximum score of 80 points and a minimum of 20 points.

##### Depressed mood and social inhibition/type D behavior

A person with Type D personality tends to worry, to feel tense and unhappy. He/she often gets irritated and seldom sees the bright side of life. He/she is also socially and emotionally inhibited with relative social isolation as a consequence. The Type-D scale 16 (DS16) [13] was used in its short form (DS 14). Examples of statements are "I am often in a bad mood" and "I am often irritated". Five response alternatives are used and scored from 0 - 4, with a maximum score of 56 points and a minimum of 0 points. The higher score corresponds to more Type D personality.

##### Covariates

Information about marital status, number of children, smoking and other health habits, type of work, and employment status was obtained through interview. A self reported history of obesity, diabetes, hypertension, hyperlipidemia, family history, diagnosis, and medication was collected.

##### Statistical Methods

To determine statistically significant effects, paired t-tests were used to compare mean scale scores before and after the intervention. SAS (Software Analytics Systems) version 8.02 was used for analyses.

## Results

In this feasibility study we present both quantitative and qualitative results. We report men and women separately and together and we present their change over time.

### Medical characteristics

The mean age of the investigated group (n = 30) was 55 ± 7 years, the mean age of women (n = 11) was 57 ± 8 and that of men (n = 19) 55 ± 6 years.

Most of the participants were cohabiting (83%) and working (68%). 55% were smoker/former smoker; most patients had the diagnoses AMI (93%; angina: 7%) and Diabetes (86%); 28% had high blood pressure. Most patients were on: ASA (100%), beta Blocker (96%), and on Statins (90%) medication. There were four participants who dropped out after beginning of the interventions program, two men and two women.

Depression scores for men, women, and the whole group improved, however not statistically significantly. Depression scores, on average, were low at baseline, both for women and in men.

In contrast, anxiety diminished during the intervention for the whole group. For women, anxiety scores decreased significantly (9. 5 ± 5.0 to 6.6 ± 4.9, p = 0.002). The change was substantial, comprising about 30 percent of the total score. Similarly for men, anxiety scores also decreased significantly (9.2 ± 4.4 to 7.7 ± 4.5, p = 0.04), but the net change was smaller, about 20 percent (Table 1).

**Table 1:** Psychosocial factors for the whole intervention group

	Before intervention			After intervention		
	**n**	**mean**	**SD**	mean	SD	p

Type A Behavior (20-80)	30	47,4	9,3	40,7	8.6	< 0,0001

Type D Behavior (0-56)	30	27,9	10,0	23,2	8.9	0,004

Sense of Coherence **(13-91)**	30	54,2	6,9	57,4	6.6	0.01

**HAD scale**						

Depression (0-23)	30	5,6	3,8	5,1	4,2	n.s.

Anxiety (0-23)	30	9,2	4,5	7,4	4,6	0,0006

**Ladder of** **Life**						

Life now (1-10)	30	5.9	2,1	6,9	2,0	0,008

Life in the past (1-10)	30	5,6	2,5	5,3	2,5	n.s

Life in the future (1-10)	30	7,8	1,8	8,0	1,3	n.s.

The intervention was accompanied by a positive effect on coronary prone behavior types both on Type A and Type D. The Type A behavior for men decreased from 46. 1 ± 9.4 to 40.1 ± 9.6 (p = 0,004) and Type A for women decreased from 49.5 ± 9.1 to 40.4 ± 7.1 (p = 0.001).

The Type D behavior for men decreased from 26.7 ± 9.3 to 22.3 ± 8.8 (p = 0.04) and Type D for women decreased from 29.9 ± 11.3 to 24.7 ± 9.2 (p = 0.04).

In summary, depressive, anxious, and stress symptoms decreased more in women than in men. The changes were statistically significant - except for depression (Table 2 and Table 3).

**Table 2:** Psychosocial factors for men

	Before intervention			After intervention		
	**n**	**mean**	**SD**	mean	SD	p

Type A Behavior (20-80)	19	46,1	9,4	40,1	9,6	0,004

Type D Behavior (0-56)	19	26,7	9,3	22,3	8,8	0,04

Sense of Coherence **(13-91)**	19	54,3	7,4	57,5	5,6	0,04

**HAD scale**						

Depression (0-23)	19	6,3	3,8	5,8	4,4	n.s.

Anxiety (0-23)	19	9,2	4,4	7,7	4,5	0,04

**Ladder of** **life**						

Life now (1-10)	19	5,7	2,1	6,8	2,1	0,03

Life in the past (1-10)	19	5,6	2,7	5,8	2,6	n.s.

Life in the future (1-10)	19	7,5	2,0	8,1	1,4	n.s.

**Table 3:** Psychosocial factors for the women

	Before intervention			After intervention		
	**n**	**mean**	**SD**	**mean**	**SD**	**p**

Type A Behavior (20-80)	11	49,5	9,1	40,4	7,1	0,001

Type D Behavior (0-56)	11	29,9	11,3	24,7	9,2	0,04

Sense of Coherence **(13-91)**	11	53,9	6,2	57,2	8,3	n.s.

**HAD scale**						

Depression (0-23)	11	4,5	3,6	3,8	3,5	n.s.

Anxiety (0-23)	11	9,5	5,0	6,6	4,9	0,002

**Ladder of** **life**						

Life now (1-10)	11	6,3	2,1	7,0	1,8	n.s.

Life in the past (1-10)	11	5,6	2,1	4,5	2,0	n.s.

Life in the future (1-10)	11	5,6	2,1	4,5	2,0	n.s.

The health related quality of life, visualized as the ladder of life, improved statistically significantly for the whole group and for the men, however not for the women.

The increase of the other two quality of life measures, ladder of life (future) and ladder of life (past), were not statistically significant either for the whole group, men, or women. The ladder of life (past) was not statistically significant for the whole group nor for men, but was for the women (8.4 ± 1.3 to 7.9 ± 1.1, p = 0.01).

Finally, the improvement in Sense of Coherence for the whole group was statistically significant. It was also statistically significant for the men (54. 3 ± 7.4 to 57.5 ± 5.6, p = 0.04), but not for the women.)

## Discussion

We have described a cognitive, behavioral intervention program tailored for patients who are recovering from an acute clinical coronary event. We have shown that it can be implemented, and we have observed that it can be perceived as meaningful by both men and women patients. We have also shown that psychosocial stress measures decrease along with the implementation process. We have not shown, however, that the program leads to a relief of stress. Although unlikely, the improvement of anxiety, depressive symptoms, social isolation, and daily stress symptoms could be the consequence of spontaneous changes occurring as time passes after a heart attack. To exclude this possibility we would have needed a control group that was simultaneously monitored. However, using the same intervention program for a different set of women patients we have previously shown that this program was accompanied by a threefold decrease in mortality from all causes and an almost equally strong effect on CVD mortality.

In that study, female patients were randomized to usual cardiological care only or to usual care combined with the behavioral intervention program. The differences between the two groups of patients were substantial. The mortality in the intervention group was about one third of the mortality in the control group [8].

Furthermore the evaluation scales that we worked with are well known and frequently used. The HADS is such an example. It is known that changes in the HADS scale scores occur in normally healthy people just because time passes; e.g. in ongoing and in recently published studies we have seen that the scores of the anxiety subscale of the HADS, decline with the passage of time [14], whereas the depression subscale seems to be more stable [15].

It is also noteworthy that although the differences were relatively modest, the effects on the psychosocial parameters of women were larger than those of men. Female patients had higher anxiety scores to begin with. Even after controlling for differences in baseline values, however, the change in the scores of women included in the study tended to be higher than those of the men.

It is generally known that women tend to report more and stronger symptoms of illness, pain, or other disease manifestation than do men. It is also generally known that more men than women die prematurely and unexpectedly; e.g. in the context of sudden cardiac death [5]. This sudden cardiac death often strikes like a lightening bolt out of the blue sky, without any premonitory symptoms at all. Before age 65 it is three to five times more common in men than it is in women. Possibly, if men were able to observe and report their suspected cardiac symptoms more readily, many sudden cardiac deaths could be prevented. In contrast, it is also possible that women over report and overemphasize their general cardiac symptoms.

### How can such gender differences be explained?

Is it possible that men would be more prone to deny their symptoms? Is it possible that men have more and stronger barriers to express their feelings and perceptions, or is it possible that men have a higher threshold for pain than women? Both explanations seem to be valid. These gender differences have not yet been fully elucidated.

Other research has shown that this same intervention method could be successfully implemented in younger female coronary patients, who were enrolled in a randomized clinical trial that met the general criteria of pharmacotherapeutic research. As mentioned above, out of those women who were randomized to receive both general cardiovascular care, including current post infarct medications, and the cognitive intervention, only eight women died over the full nine year follow up period, whereas in the usual care group, 25 women died during the same time(7 vs. 20%, p = 0.001) [8]. Most of these female patients were in their sixties, the mean age being 62 +/-, so they still had one or two decades of reasonably vital and healthy life before them. Furthermore, the women who received both the cognitive education and medication with statins, the latest and most successful contribution to pharmacotherapeutic secondary prevention, had the very lowest mortality. Only one out of 65 women with such treatments died. This was less than one percent over the follow up period.

In a prior study that enrolled almost a thousand younger male patients, the original form of this method was applied and tested. Although the method was originally intended to dampen and treat a full blown coronary prone so called Type A behavior, its usefulness seems to go much further than this kind of multifaceted behavioral syndrome.

In San Francisco men of about the same age as our Stockholm women, this method was shown to attenuate their behavior (characterized by free floating hostility etc). However, more important, it also statistically significantly decreased all cause mortality [5].

Together these studies provide evidence that behavioral interventions may reduce stress and anxiety and that they may improve both the subjective well-being and increase survival in women, as in men. In the present study we suggest that the same method is best implemented with men and women in separate groups. The program content was the same, but the origin and nature of stressors differed between men and women. We therefore suggest that cognitive behavioral interventions be performed in gender segregated groups.

## Competing interests

The author declares that they have no competing interests.

## References

[B1] DeBacker G Ambrosioni E Borch-Johnsen K Brotons C Cifkova R Dallongeville J European guidelines on cardiovascular disease prevention in clinical practice: third joint task force on European and other societies on cardiovascular disease prevention in clinical practice (constituted by representiatives of eight societies and by invited experts) Eur J Cardiovasc Prev Rehab 2003 10 4 S1 S10 10.1097/00149831-200308000-00004 14555889

[B2] Dusseldorp E van Elderen T Maes S Meulman J Kraaij V A meta-analysis of psychoeducational programs for coronary heart disease patients Health Psychology 1999 18 5 506 19 10519467 10.1037//0278-6133.18.5.506

[B3] Linden W Stossel C Maurice J Psychosocial interventions for patients with coronary artery disease: a meta-analysis Arch Intern Med 1996 156 7 745 52 10.1001/archinte.1996.00440070065008 8615707

[B4] Jackson L Leclerc J Erskine Y Linden W Getting the most out of cardiac rehabilitation: a review of referral and adherence predictors Heart 2005 91 1 10 4 10.1136/hrt.2004.045559 15604322 PMC1768637

[B5] Friedman M Thoresen CE Gill JJ Ulmer D Powell LH Price VA Alteration of Type A behavior and its effect on cardiac recurrences in post myocardial infarction patients: Summary results of the Recurrent Coronary Prevention Project Am Heart J 1986 112 653 65 10.1016/0002-8703(86)90458-8 3766365

[B6] Burell G Granlund B Women's hearts need special treatment Int J Behav Med 2002 9 228 42 10.1207/S15327558IJBM0903_05 12360839

[B7] Orth-Gomér K Moser V Blom M Wamala SP Schenck-Gustafsson K Kvinnostress kartläggs: Hjärtsjukdom hos Stockholmskvinnor orsakas i lika hög grad av stress i familjen som i arbetet Läkartidningen (Journal of the Swedish Medical Association) 1997 94 8 632 8 9072654

[B8] Orth-Gomér K Schneiderman N Wang HX Walldin C Blom M Jernberg T Stress reduction prolongs life in women with coronary disease: the Stockholm Women's Intervention Trial for Coronary Heart Disease (SWITCHD) Circ Cardiovasc Qual Outcomes 2009 2 1 25 32 10.1161/CIRCOUTCOMES.108.812859 20031809

[B9] Blom M Georgiades A Janszky I Alinaghizadeh H Lindvall B Ahnve S Daily stress and social support among women with CAD: results from a 1-year randomized controlled stress management intervention study Int J Behav Med 2009 16 3 227 35 10.1007/s12529-009-9031-y 19277873

[B10] Antonovsky A The life cycle, mental health and sense of coherence Isr J Psychiatry Relat Sci 1985 22 4 273 80 3836223

[B11] Zigmond AS Snaith RP The hospital anxiety and depression scale Br Med J 1986 292 6516 344 3080166 10.1136/bmj.292.6516.344 PMC1339318

[B12] Yarnold PR Mueser KT Grau BW Grimm LG The reliablility of the student version of the Jenkins Activity Survey J Behav Med 1986 9 4 401 14 10.1007/BF00845123 3746906

[B13] Denollet J Personality and coronary heart disease: the type-D scale 16 (DS16) Ann Behav Med 1998 20 3 208 15 10.1007/BF02884962 9989328

[B14] Merswolken M Siebenhuener S Orth-Gomér K Zimmermann-Viehoff F Deter HC Treating anxiety in patients with coronary heart disease: a randomized controlled trial. Psychother Psychosom 2011 80 6 365 70 10.1159/000329177 21968457

[B15] Albus C Beutel ME Deter HC Fritzsche K Hellmich M Jordan J Juenger J Krauth C Ladwig KH Michal M Mueck-Weymann M Petrowski K Pieske B Ronel J Soellner W Waller C Weber C Herrmann-Lingen C A stepwise psychotherapy intervention for reducing risk in coronary artery disease (SPIRR-CAD) - rationale and design of a multicenter, randomized trial in depressed patients with CAD J Psychosom Res 2011 71 4 215 22 10.1016/j.jpsychores.2011.02.013 21911098

